# Exosomal secretion of a psychosis-altered miRNA that regulates glutamate receptor expression is affected by antipsychotics

**DOI:** 10.1038/s41386-019-0579-1

**Published:** 2019-11-27

**Authors:** Stephen K. Amoah, Brian A. Rodriguez, Constantine N. Logothetis, Praveen Chander, Carl M. Sellgren, Jason P. Weick, Steven D. Sheridan, Lauren L. Jantzie, Maree J. Webster, Nikolaos Mellios

**Affiliations:** 1 Department of Neurosciences, University of New Mexico School of Medicine, Albuquerque, NM USA; 2 Autophagy inflammation and metabolism (AIM) center, Albuquerque, NM USA; 3 Department of Physiology and Pharmacology, Karolinska Institutet, Stockholm, Sweden; 4 Center for Genomic Medicine, Chemical Neurobiology Laboratory, Departments of Neurology and Psychiatry, Massachusetts General Hospital and Harvard Medical School, Boston, MA USA; 5 Center for Experimental Drugs and Diagnostics, Center for Genomic Medicine, Massachusetts General Hospital, Boston, MA USA; 6 Department of Psychiatry, Harvard Medical School, Boston, MA USA; 7 Department of Pediatrics, Johns Hopkins School of Medicine, Baltimore, MD USA; 8 Laboratory of Brain Research, Stanley Medical Research Institute, Chevy Chase, MD USA

**Keywords:** Cellular neuroscience, Post-translational modifications

## Abstract

The ability of small secretory microvesicles known as exosomes to influence neuronal and glial function via their microRNA (miRNA) cargo has positioned them as a novel and effective method of cell-to-cell communication. However, little is known about the role of exosome-secreted miRNAs in the regulation of glutamate receptor gene expression and their relevance for schizophrenia (SCZ) and bipolar disorder (BD). Using mature miRNA profiling and quantitative real-time PCR (qRT-PCR) in the orbitofrontal cortex (OFC) of SCZ (*N* = 29; 20 male and 9 female), BD (*N* = 26; 12 male and 14 female), and unaffected control (*N* = 25; 21 male and 4 female) subjects, we uncovered that miR-223, an exosome-secreted miRNA that targets glutamate receptors, was increased at the mature miRNA level in the OFC of SCZ and BD patients with positive history of psychosis at the time of death and was inversely associated with deficits in the expression of its targets glutamate ionotropic receptor NMDA-type subunit 2B (*GRIN2B*) and glutamate ionotropic receptor AMPA-type subunit 2 (*GRIA2*). Furthermore, changes in miR-223 levels in the OFC were positively and negatively correlated with inflammatory and GABAergic gene expression, respectively. Moreover, miR-223 was found to be enriched in astrocytes and secreted via exosomes, and antipsychotics were shown to control its cellular and exosomal localization in a cell-specific manner. Furthermore, addition of astrocytic exosomes in neuronal cultures resulted in a significant increase in miR-223 expression and a notable reduction in *Grin2b* and *Gria2* mRNA levels, which was strongly inversely associated with miR-223 expression. Lastly, inhibition of astrocytic miR-223 abrogated the exosomal-mediated reduction in neuronal *Grin2b* expression. Taken together, our results demonstrate that the exosomal secretion of a psychosis-altered and glial-enriched miRNA that controls neuronal gene expression is regulated by antipsychotics.

## Introduction

Schizophrenia (SCZ) and bipolar disorder (BD) are heterogeneous psychiatric disorders that manifest during late adolescence and young adulthood, and together affect ~3.5% of the US adult population [1, 2]. Pharmacological, genetic, postmortem, and animal studies have linked N-methyl-d-aspartate receptor (NMDA) hypofunction [3, 4], impairments in dopamine [4–6], wingless-related integration site (WNT) [7, 8], or calcium signaling [9], and GABAergic gene expression deficits [10–15] to SCZ and/or BD. Moreover, an increasing number of studies have reported an upregulation in non-neuronal gene expression related to inflammation in the brain of subjects with SCZ [16–22] and less in BD [23], such as the acute-phase inflammation marker serpin family A member 3 (*SERPINA3*—also known as alpha 1-antichymotrypsin) [16, 19, 21].

MicroRNAs (miRNAs) are a subcategory of small evolutionarily conserved noncoding RNAs (ncRNAs) ~18–22 nucleotides in length [24, 25]. They are transcribed independently as large precursor RNA molecules from intergenic regions or spliced from introns of protein-coding genes and are subsequently cleaved into mature miRNA transcripts [24–26]. Numerous studies have suggested that miRNAs post-transcriptionally control gene expression by binding to complementary sequences in the 3′ untranslated region (UTR) of mRNAs, thereby inhibiting mRNA translation and leading to subsequent mRNA decay [24, 27]. In the brain, miRNAs display a developmental-, laminar-, and cell-specific expression and can modulate molecular functions ranging from neurogenesis, neuronal differentiation, and circuitry establishment, to plasticity [28–31].

Recent work has revealed the presence of small secretory microvesicles between 30 and 150 nm, known as “exosomes”, which are mainly produced from the multivesicular bodies of the endosomal pathway [32, 33]. Exosomes contain both proteins and RNAs of the cell of origin and are particularly enriched in miRNAs [32–35]. Exosomes can fuse with the plasma membrane of the recipient cells and unload their miRNA cargo, which in turn facilitates the post-transcriptional inhibition of multiple protein-coding genes [34, 35]. The secretion of large numbers of exosomes from glia and neurons, and the perceived ability of exosomes to influence various aspects of neuronal and glial development and function, has positioned them as an exciting and novel method of cell-to-cell communication with important implications for brain disease [33–35].

Despite the numerous studies showing miRNA dysregulation in postmortem brains of subjects with psychiatric disorders [14, 36–44], and the relevance of miRNA-processing genes for SCZ [45–47], very little is known about the role of exosome-associated miRNAs in SCZ and BD, their interactions with neuronal gene expression, and their response to treatment. Here we show that miR-223, a miRNA known to be secreted via exosomes [48, 49], is significantly increased in the orbitofrontal cortex (OFC) of subjects with SCZ and in BD patients with psychosis at the time of death, is positively correlated to *SERPINA3* expression, and negatively associated with its targets [50] glutamate ionotropic receptor α-amino-3-hydroxy-5-methyl-4-isoxazolepropionic acid (AMPA)-type subunit 2 (*GRIA2*, also known as *GLUR2*) and glutamate ionotropic receptor NMDA-type subunit 2B (*GRIN2B*, also known as *NR2B*). Moreover, miR-223 is enriched within astrocytes and highly expressed in glial and neuronal exosomes and is differentially regulated by antipsychotic treatment in mouse cortical neuronal and astrocytic cultures. Lastly, addition of astrocytic exosomes in cortical neurons results in increased neuronal miR-223 expression and reductions in *Grin2b* mRNA levels, which are rescued following inhibition of miR-223 in astrocytes. Taken together, our data suggest that a psychosis-altered and glial-enriched miRNA, whose expression could be regulated by antipsychotics, is secreted by exosomes in order to inhibit neuronal NMDA receptor gene expression.

## Materials and methods

### Animal experiments

The Institutional Care and Use Committee (IACUC) at the University of New Mexico Health Sciences Center approved all experimental procedures (protocol No: 17-200657-HSC). For each experiment described, equal numbers of male and female pups were used, and data represent true *n* (individual pups).

### Postmortem samples

Human postmortem brain total RNA samples from the OFC of subjects with SCZ (*N* = 29), BD (*N* = 26), and unaffected controls (*N* = 25) were derived from the Stanley Medical Research Institute [51]. Samples with an RNA integrity number (RIN) higher than 6.5 were selected for RNA quantification. Summarized and detailed demographics are shown in Tables S1–2.

### RNA extraction and mRNA/miRNA quantification

RNA extraction and mRNA/miRNA quantification was done as shown before [52–55]. Briefly, total RNA was isolated using the miRNeasy RNA isolation kit (Qiagen, Hilden, Germany). RNA quality and concentration were assayed through Nanodrop 2000 spectrophotometer and Qubit 3 (ThermoFisher Scientific, Waltham, MA). Mature miRNA reverse transcription was performed with Taqman miRNA Reverse Transcription kit and quantitative real-time PCR (qRT-PCR) was done using Taqman miRNA assays (all from ThermoFisher Scientific). All miRNA qRT-PCR measurements were performed in triplicate for each sample and the mean of the three cycle thresholds (Cts) was calculated. Two miRNAs highly expressed in the OFC that are not altered in SCZ and BD (miR-30d-5p and let-7e) based on our own miRNA NanoString profiling (see also Supplementary methods and materials) were used for miRNA normalization via their geometric mean (Tables S3–4). For miRNA quantification, the following formula was used: Relative value = 2^Ct^geometric mean of miR-30d and let-7e^/2^Ct^miRNA^. In cases where no normalization was used, the following formula was utilized: Relative value = 2^–Ct^miRNA^. For mRNA quantification, 100–400 ng of total RNA was reverse transcribed using the SuperScript IV First-Strand Synthesis System (ThermoFisher Scientific). cDNA was then used together with Taqman mRNA primers (ThermoFisher Scientific) for mRNA qRT-PCR. *18S rRNA* was used as a normalizer in cDNA samples further diluted by 20-fold and showed no changes in either BD or SCZ OFC relative to controls (Fig. S1). For mRNA quantification, the following formula was used: Relative value = E^Ct^normalizer^/E^Ct^mRNA^, where E = 10^(−1/primer slope). Detailed information about the Taqman mRNA, miRNA, and pri-miRNA primers used in our study is included in Table S5.

## Results

### Significant associations between changes in miR-223 and glutamate receptor, GABAergic, and inflammatory gene expression in the OFC of subjects with psychiatric disorders

**Fig. 1:**
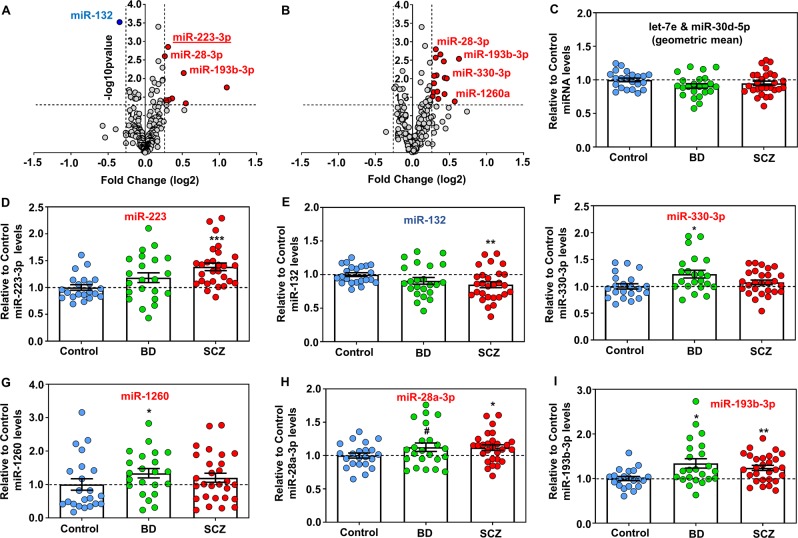
Dysregulation of miRNA expression in SCZ and BD OFC. **a**, **b** Results from NanoString nCounter miRNA profiling from the 200 most highly expressed miRNAs in the OFC presented as a volcano plot. The *x* axis represents log2 fold changes and the *y* axis represents the *p*-value with 1.2-fold and *p* < 0.05 cutoff, respectively. Significantly more than 1.2-fold increased miRNAs are shown as red circles and significantly more than 1.2-fold downregulated as blue circles. Notice the significant elevation of several miRNAs in SCZ (**a**) and BD (**b**) with one miRNA reduced in SCZ. Altered miRNAs chosen to be validated with qRT-PCR are named in the graph. **c**–**i** Graphs showing mean ± SEM relative to the mean of unaffected controls let-7e and miR-30d-5p geometric mean (**c**), miR-223-3p (**d**), miR-132 (**e**), miR-330-3p (**f**), miR-1260 (**g**), miR-28-3p (**h**), and miR-193b-3p (**i**) levels in SCZ, BD, and controls based on mature miRNA qRT-PCR. Graphs in (**d**–**i**) are normalized to the geometric mean of unaltered in BD and SCZ miRNAs let-7e and miR-30d-5p (see also (**c**) and Materials and methods). Data from each case are also depicted in the graph as blue circles (control), green circles (BD), and red circles (SCZ). ^#^0.05 < *p* <0.10, *p < 0.05, **p < 0.01, ***p < 0.001, based on a univariate general linear model that corrects for RIN, brain pH, PMI, and refrigeration interval.

We utilized postmortem brain samples from the OFC of subjects with SCZ (*N* = 29), BD (*N* = 26), and unaffected controls (*N* = 25) derived from the Stanley Medical Research Institute [51]; only samples with an RNA integrity number (RIN) higher than 6.5 were included (Tables S1–2). To selectively and accurately detect mature miRNA transcripts we utilized NanoString nCounter miRNA profiling (Tables S3–4). Focusing on the top 200 most highly expressed miRNAs in the OFC, we uncovered a subset of dysregulated SCZ and/or BD miRNAs (Fig. 1a, b and Tables S3–4). Using mature miRNA qRT-PCR, with the geometric mean of two highly expressed miRNAs that are not altered in SCZ and BD OFC as a normalizer (miR-30d-5p and let-7e, based on both the miRNA NanoString and qRT-PCR—Fig. 1c and Tables S3–4), and further correcting all results for RIN, brain pH, postmortem interval, and refrigeration interval using a univariate general linear model (see also “Materials and methods” section), we first validated increases in the majority of cases with SCZ but not in BD in miR-223-3p (mentioned henceforth as miR-223), a miRNA shown to target NMDA and AMPA (AMPA) receptor subunits, and thus, regulate rodent and human neuronal function [50, 56] (Fig. 1d). On the other hand, miR-132, a neuronal-enriched miRNA involved in synaptic plasticity [54] was reduced in SCZ (Fig. 1e), in accordance with previous reports [41]. Moreover, miRNA-specific qRT-PCR confirmed moderate increases in the expression of both miR-330-3p and miR-1260 in BD (Fig. 1f, g). Lastly, we validated an increase in miR-193b-3p and miR-28a-3p in both SCZ and BD (Fig. 1h, i). Although a subset of these altered miRNAs have been reported to be secreted by exosomes, only miR-223 is a bona fide exosome-enriched miRNA shown in numerous reports to control glial and immune cell gene expression in response to ischemia and neuroinflammation [48, 49, 56–58]. Furthermore, a previous study reported that olanzapine treatment can significantly reduce cortical levels of miR-223 in adult mice [59], thus suggesting that it could also be responsive to antipsychotics.

**Fig. 2:**
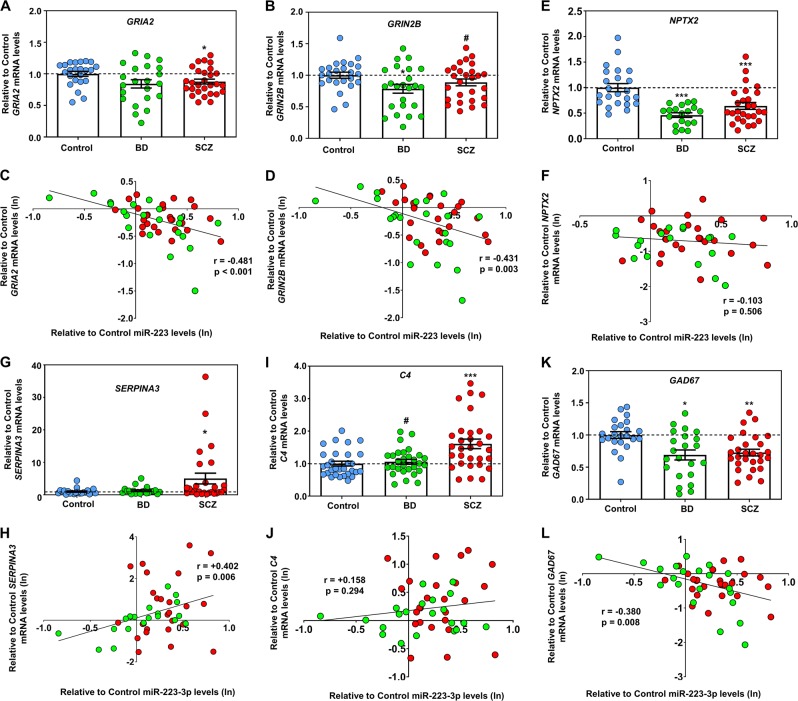
Alterations in glutamate receptor subunit, GABAergic, and inflammatory gene expression in the OFC are significantly associated with miR-223 changes in SCZ and BD. **a, b, e** Graphs showing mean ± SEM relative to the mean of unaffected controls mRNA levels in SCZ, BD, and control OFC for *GRIA2* (**a**), *GRIN2B* (**b**), and *NPTX2* (**e**) mRNAs, based on qRT-PCR and normalized to the unaltered in SCZ and BD 18S rRNA (see also Fig. S1 and Materials and methods). **c**–**d**, **f** Correlations between changes in miR-223 and *GRIA2* (c), *GRIN2B* (**d**), and *NPTX2* (**f**) mRNA expression in the OFC of subjects with SCZ and BD. Spearman’s correlation coefficients and two-tailed *p*-values are shown in the graphs. **g**, **i**, **j** Graph showing mean ± SEM relative to the mean of unaffected controls mRNA levels in SCZ, BD, and control OFC for *SERPINA3* (**g**), *C4* (i), and *GAD1* (**k**) based on qRT-PCR and normalized to *18S rRNA*. **h**, **j**, **l** Correlations between changes in miR-223 and *SERPINA3* **(h)**, *C4* (**j**), and *GAD1* (**l**) expression in the OFC of subjects with SCZ and BD. Data from each case are also depicted in the graph as blue circles (control), green circles (BD), and red circles (SCZ). Spearman’s correlation coefficients and two-tailed *p*-values are shown in the graphs. For (**a**–**c**, **g**–**i**) ^#^0.05 < *p* < 0.10, *p < 0.05, **p < 0.01, ***p < 0.001, based on a univariate general linear model that corrects for RIN, brain pH, PMI, and refrigeration Interval. Data from each case are also depicted in the graph as blue circles (control), green circles (BD), and red circles (SCZ).

Focusing on miR-223, we then examined the expression of proven miR-223 targets, *GRIA2* and *GRIN2B* [50], which are of relevance to psychiatric disorders, in our cohort using qRT-PCR with normalization to the unaltered and reliable for postmortem studies *18S rRNA* [14, 53, 60] (Fig. S1). Our results, which were again further corrected for multiple postmortem demographics using a univariate general linear model, showed a significant reduction in *GRIA2* mRNA and a trend for reduction in *GRIN2B* mRNA in the OFC of subjects with SCZ, with *GRIN2B* also being downregulated in BD (Fig. 2a, b). Moreover, changes in miR-223 levels in the OFC of subjects with SCZ/BD were significantly inversely correlated with *GRIA2* and *GRIN2B mRNA* expression (Fig. 2c, d). These correlations were specific, since other significantly decreased SCZ/BD mRNAs produced by neurons, such as neuronal pentraxin 2 (*NPTX2*) [61] (a gene that is not upstream or downstream of miR-223), did not show any association with miR-223 (Fig. 2e, f). Moreover, changes in astrocyte-enriched inflammation-related mRNA *SERPINA3* were positively associated with miR-223 expression (Fig. 2g, h). Again, this positive correlation with increased *SERPINA3* in SCZ appeared to be specific, and no association was found with the expression of other inflammation-related genes known to be increased in psychiatric disorder postmortem brains, such as Complement 4 (*C4*) [62] (Fig. 2i, j). Of note, we did observe a modest negative correlation between miR-223 and changes in *GAD1* mRNA, which is not a target of miR-223, suggesting a potential indirect association with GABAergic gene expression (Fig. 2k, l). We therefore conclude that alterations in exosome-enriched miR-223 in the OFC of subjects with psychiatric disorders are strongly associated with changes in miR-223 targets related to glutamate receptor gene expression.

Analyzing the primary miR-223 precursor transcript, pri-miR-223, we observed that there was no significant difference, rendering transcriptional alterations as an unlikely source of the upregulation in mature miR-223 transcript levels seen in SCZ (Fig. S2a). In contrast, pri-miR-132 appeared to be reduced in SCZ, suggesting a transcriptional mechanism for the observed deficits in mature miR-132 expression (Fig. S2b). We then assessed the levels of genes involved in miRNA processing. We found no changes in our cohort in *DICER1*, and DiGeorge syndrome chromosomal region 8 (*DGCR8*) (Fig. S2c–d), in contrast to what has been observed in other brain regions in SCZ [37, 63]. We did observe, though, that both adenosine deaminases acting on RNA transcript types 1 and 2 (*ADAR1*, *ADAR2*) were significantly downregulated in SCZ, with *ADAR2* also showing a modest reduction in BD (Fig. S2e–f). ADARs are deaminases that convert adenosine to inosine, resulting in reduced pri-miRNA processing and/or degradation of intermediate precursor (pre-miRNA) transcripts, both of which ultimately result in reduced mature miRNA levels [64, 65]. We analyzed the relationship between miR-223, and *ADAR1* and *ADAR2*, and observed that there was a significant inverse correlation between miR-223 and ADAR1 and ADAR2, with the association with ADAR2 being the strongest (Fig. S2g–h). Taken together, our results indicate that reduced *ADAR1/2* levels are associated with increased expression of mature miR-223 in SCZ. These data suggest that dysregulation of mature miR-223 expression in the OFC of subjects with SCZ is unlikely to be a result of altered pri-miRNA transcription or canonical miRNA processing, but instead appears to be associated with reduced *ADAR*-mediated inhibition of miRNA processing.

### Specific upregulation of miR-223 in the OFC of BD subjects with psychosis

**Fig. 3:**
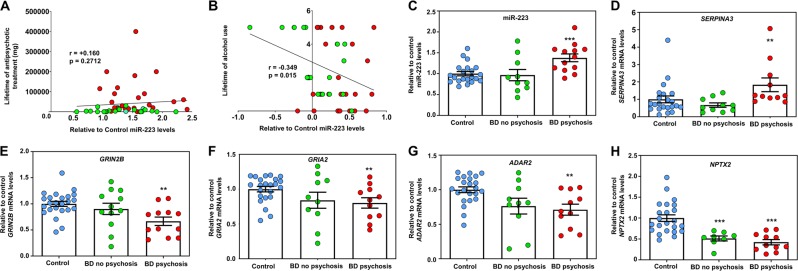
BD patients with psychosis display altered OFC miR-223 and miR-223-associated gene expression similar to that seen in SCZ. **a**, **b** Correlation between relative-to-control changes in miR-223 and lifetime of antipsychotic treatment (**a**; shown as mg of fluphenazine equivalents) and alcohol use (**b**) in the OFC of subjects with SCZ and BD. Spearman’s correlation coefficients and two-tailed *p*-values are shown in the graph. Relative-to-control individual data are also shown in the graphs: SCZ = red circles, BD = green circles. **c**–**h** Graphs showing mean ± SEM relative to the mean of unaffected controls mRNA levels in BD with no psychosis, BD with psychosis, and control OFC for miR-223 (**c**), *SERPINA3* (**d**) *GRIN2B* (**e**), *GRIA2* (**f**), *ADAR2* (**g**), and *NPTX2* (**h**) expression based on qRT-PCR (mRNA expression normalized to *18S rRNA* and miRNA expression to the geometric mean of let-7e and miR-30d). Data from each case are also depicted in the graph as blue circles (control), green circles (BD with no psychosis), and red circles (BD with psychosis). *p < 0.05, **p < 0.01, ***p < 0.001, based on a univariate general linear model that corrects for RIN, brain pH, PMI, and refrigeration interval.

To determine whether postmortem demographics could be influencing miR-223 expression in the OFC, we examined the effects of 18 separate demographics on the relative-to-control changes of miR-223 in BD and SCZ OFC (Table S6). We detected no interactions with most of the postmortem demographics, including duration of overall antipsychotic treatment (shown as fluphenazine equivalents in mg) (Table S6 and Fig. 3a). However, we observed a negative correlation between changes in miR-223 and lifetime of alcohol use (which could not account for the observed increases in miR-223 expression given the higher alcohol use in patients vs. controls) and a significant positive association with psychosis at the time of death (Table S6 and Fig. 3b). Although all SCZ patients are positive for psychosis, only a subset of subjects with BD in our cohort were diagnosed with psychosis close to the time of death (Table S2). We therefore separated the BD group into BD with and without psychosis at the time of death. We found that miR-223 expression was significantly increased in BD patients with psychosis, whereas BD subjects without psychosis showed similar-to-control values (Fig. 3c). Given the observed significant correlations between miR-223 and *SERPINA3*, *GRIN2B*, *GRIA2*, and *ADAR2* mRNAs in the OFC, we plotted their expression into each of the two BD groups (Fig. 3d–g). Our results showed that BD patients with psychosis but not BD patients without psychosis displayed significant increases in *SERPINA3*, and reductions in *GRIN2B*, *GRIA2*, and *ADAR2* mRNAs, similar to those seen in SCZ (Fig. 3d–g). Of note, *NPTX2* mRNA, which is not associated with miR-223, was seen to be equally reduced in both BD patients with psychosis and without psychosis at the time of death (Fig. 3h). We conclude that changes in OFC miR-223 and miR-223-associated gene expression are observed in BD patients with psychosis similar to what is seen in SCZ.

### miR-223 is enriched in astrocytes and exosomes and is regulated by antipsychotics in a cell-specific manner

**Fig. 4:**
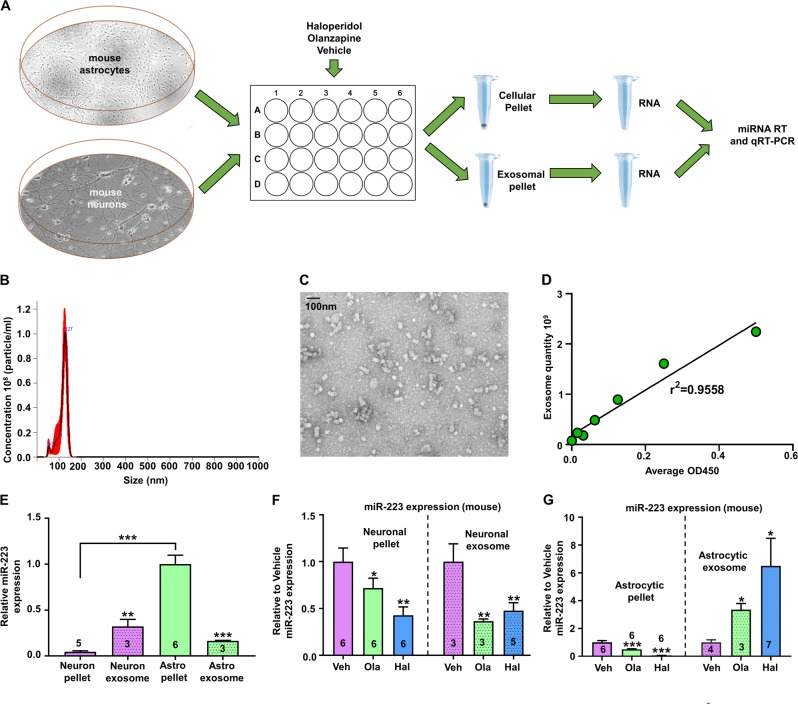
Enrichment of miR-223 in astrocytes and cell-specific effects of antipsychotic treatment on miR-223 expression exosomal secretion. **a** Experimental design. **b**–**d** Exosome quantification with NanoSight (**d**), electron microscopy characterization (**c**), and dilution curve with plate reader based on ELISA CD63 exosome marker reactivity (**d**). **e** Mean ± SEM miR-223 relative expression in mouse cortical neuronal (2 weeks of differentiation) and astrocytic pellets and exosomes (based on mature miRNA qRT-PCR without normalization and shown as ratios relative to the highest expression in mouse astrocytic pellets). **p < 0.01, ***p < 0.001, based on two-tailed one- sample *t* test compared with pellet expression of the same cell culture (stars above bars) or compared with pellet expression between different cell types (stars with connecting line). **f**, **g** Mean ± SEM miR-223 levels (based on mature miRNA qRT-PCR, without normalization, and normalized to the mean of either pellet or exosomal vehicle) in primary mouse cortical neuronal (grown in culture for 18 days) (**f**) and astrocytic (**g**) pellets and exosomes treated for 2 days with olanzapine (Ola), haloperidol (Hal), or vehicle (Veh). *p < 0.05, **p < 0.01, ***p < 0.001, based on two-tailed one-sample *t* test compared with the mean of miR-223 expression in vehicle-treated cultures of the same isolation (pellet or exosome). The number of biological replicates is shown in each graph.

Although the enrichment of miR-223 in peripheral exosomes has been previously studied (48–50, 57), little is known about its expression in the brain. To that end, we compared its cellular and exosomal expression in primary mouse cortical and astrocytic cultures (Fig. 4a). We utilized various techniques to detect and quantify exosomes. Exosomal quantity was determined using nanoparticle-tracking analysis via NanoSight (NS300) (Fig. 4b), electron microscopy was used to visualize the size of the exosomes (Fig. 4c), and ELISA via CD63 immunoreactivity to quantity serial dilutions of exosomes, which indicated a robust linear correlation (Fig. 4d). We next analyzed the expression of miR-223 in the cellular pellets and exosomal fractions of mouse cortical neurons and astrocytes. We found that the cellular expression of miR-223 was significantly higher in astrocytes than in neurons, whereas exosomes from both neurons and astrocytes expressed equal amounts of miR-223 (Fig. 4e). Conversely, analysis of let-7e expression in mouse cortical neurons and astrocytes showed an enrichment in neuronal pellets with lower levels in the exosomal fractions (Fig. S3a).

Given that previous work has indicated that olanzapine reduces miR-223 expression in the mouse brain [59], we were interested in the effect of the antipsychotics on cellular and exosomal miR-223 levels in cortical neurons and astrocytes. Using a subacute (2-day) treatment of olanzapine and haloperidol, we found that antipsychotics significantly reduce miR-223 expression in both neuronal pellets and neuronal exosomal fractions (Fig. 4f), suggesting a reduction in miR-223 synthesis within neurons. On the other hand, subacute treatment of the same antipsychotics reduced miR-223 expression in astrocytic pellets but increased miR-223 levels in the exosomal fraction, suggesting that antipsychotics could increase the secretion of miR-223 in astrocytes (Fig. 4g). Previous work has shown that miR-223 is not expressed in adult mouse microglia yet is abundant in mouse macrophages or monocytes [66]. To determine miR-223 expression in human microglia and monocytes/macrophages, we pulled NanoString profiling data for miR-223 from a previous study [67]. These results showed that although miR-223 was barely detected in purified human adult brain microglia, there was robust expression of miR-223 in peripheral monocytes and macrophages (Fig. S4). We conclude that in the brain parenchyma, miR-223 is enriched in mouse astrocytes and secreted via exosomes, and that antipsychotics reduce miR-223 synthesis in neurons but increase miR-223 exosomal secretion in astrocytes.

### Mouse astrocytic exosomes assimilated by rat neurons increase miR-223 levels and downregulate miR-223-associated downstream targets

**Fig. 5:**
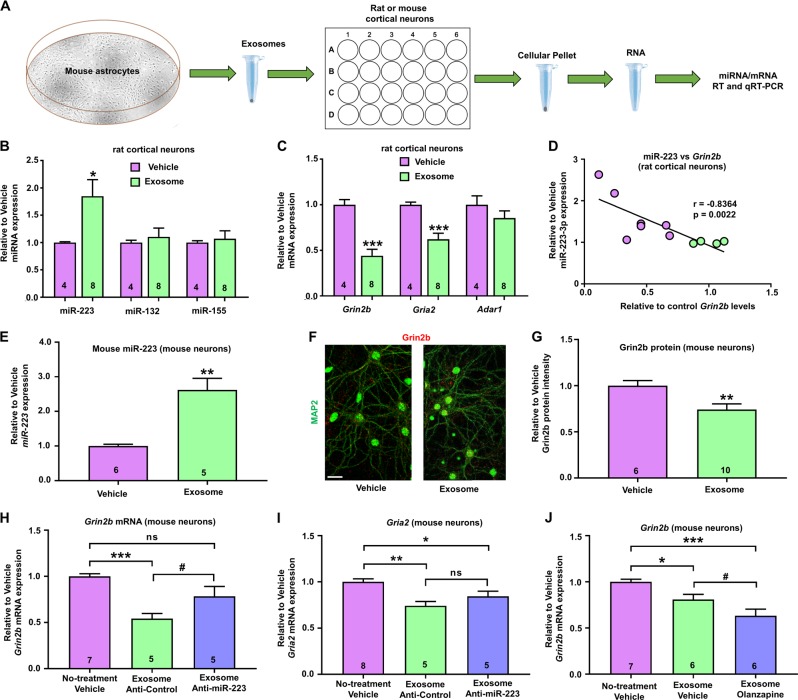
Astrocytic exosomes increase neuronal miR-223 levels and downregulate miR-223-related neuronal gene expression. **a** Schematic of experimental design. **b** Graph showing mean ± SEM mature miR-223, miR-132, and miR-155 expression (based on mature miRNA qRT-PCR and normalized to highly expressed and unaltered let-7e—see Fig. S5a) in rat neurons treated with mouse astrocytic exosomes for 1–2 days relative to vehicle-treated rat neurons. **c** Graph showing mean ± SEM rat neuronal gene levels following mouse exosomal treatment for rat *Grin2b, Gria2*, and *Adar2* mRNAs (all based on qRT-PCR and normalized to *18S rRNA*—see Fig. S5b). Data are shown as ratios relative to the mean of vehicle. **d** Correlation between relative-to-vehicle miR-223 (mouse primer) and rat *Grin2b* mRNA expression. Spearman’s correlation coefficients and two-tailed *p*-values are shown in the graphs. Vehicle = purple circles, exosome treatment = green circles. **e** Graph showing mean ± SEM mouse mature miR-223 expression (based on mature miRNA qRT-PCR and normalized to let-7e) in mouse cortical neurons treated with mouse astrocytic exosomes for 2 days relative to vehicle-treated rat neurons. For (**b**, **c**, **e**) *p < 0.05, **p < 0.01, ***p < 0.001, based on two-tailed one-sample *t* test compared with vehicle. **f** Representative images following immunostaining of mouse cortical neurons with anti-Grin2b (red) and anti-MAP2 antibodies (green) after 2 days of treatment with astrocytic exosomes (Exosome) or no treatment (Vehicle). Scale bar = 50 µm. **g** Graph showing mean ± SEM relative to no-treatment vehicle Grin2b immunostaining intensity in mouse cortical neurons positive for MAP2 treated with mouse astrocytic exosomes for 1 day relative to vehicle-treated rat neurons (data from 3 to 4 images were averaged for each well/coverslip). **p < 0.01, based on two-tailed one-sample *t* test compared with vehicle. **h**, **i** Graphs showing mean ± SEM mouse neuronal gene levels following for mouse *Grin2b* (**h**) and *Gria2* (**i**) mRNAs (all based on qRT-PCR and normalized to *18S rRNA*) following 1 day of treatment with astrocytic exosomes treated with locked nucleic acid (LNA)-based miRNA inhibitors against mouse miR-223 (Anti-miR-223) or scrambled contol (Anti-Control)  astrocytic exosomes. **j** Graph showing mean ± SEM relative to no-treatment vehicle mouse *Grin2b* mRNA levels (based on qRT-PCR and normalized to *18S rRNA*) following 2 days of treatment with exosomes from astrocytes treated with olanzapine or vehicle, or no exosome treatment. For (**h**–**j**): ^#^0.05 < *p* < 0.10, *p < 0.05, **p < 0.01, ***p < 0.001, based on ANOVA with Tukey’s multiple comparisons test. The number of biological replicates is shown in each graph.

Given the observed enrichment of miR-223 in astrocytes and its abundance in exosomes, we decided to test whether exosomes secreted from astrocytes could regulate neuronal gene expression. Given that exosomes can also include mRNAs in addition to miRNAs, we added mouse astrocytic exosomes into rat cortical neuronal cultures so as to be certain that any changes in mRNA expression in recipient neuronal cultures are not a result of direct transport of mRNAs via exosomes. To that end, we harvested exosomes from astrocytic conditioned media and incubated the exosomes with rat cortical neurons for 1–2 days, making sure to change the media and carefully rinse the cells before harvesting the rat neuronal pellets (Fig. 5a). Using mature miRNA qRT-PCR with primers that preferentially but not exclusively detect mouse miR-223 (due to a single-nucleotide difference between mouse and rat mature miR-223 sequences, these primers are expected to detect some small percentage of rat miR-223) as well and normalizing to let-7e expression (Fig. S5a), we first observed a significant twofold increase in mature miR-223 expression in rat cortical neurons treated with mouse astrocytic exosomes as compared with untreated rat cultures (Fig. 5b). However, no changes were observed as a result of astrocytic exosome treatment in other miRNAs associated with exosomes, such as miR-132 (enriched in neuronal exosomes) and miR-155 (enriched in microglial exosomes) (Fig. 5b) [68, 69], suggesting that enrichment of miR-223 in the astrocytic exosomal fraction could enable its assimilation into the rat neuronal culture. Interestingly, qRT-PCR analysis of rat mRNA expression with rat-specific primers and normalization to *18S rRNA* (Fig. S5b) revealed a significant reduction in *Gria2* and *Grin2b,* but not *Adar1,* levels in exosome-treated rat cortical neurons, suggesting a preferential reduction in validated miR-223 targets (Fig. 5c). In order to examine whether the reductions in rat *Grin2b* and *Gria2* mRNA levels are associated with increased mouse miR-223 expression, we compared mouse miR-223 with rat and *Grin2b* and *Gria2* mRNA expression. These comparisons revealed a strong inverse correlation between miR-223 and *Grin2b* (Fig. 5d), a significant negative correlation with *Gria2* (*r* = −0.7091, *p* = 0.0182, Spearman’s correlation with two-tailed *t* test), but no significant associations between miR-223 and *Adar1* mRNA levels (data not shown). In addition, no such associations were observed between *Gria2* and *Grin2b* and miR-132 or miR-155 (Fig. S5c–f), suggesting that these changes could be driven specifically by exosomal trafficking of mouse miR-223. Of note, we also observed a trend for a negative correlation between miR-223 and reductions in rat *Gad1* mRNA (Fig. S5g–h). To make sure that the effects of astrocytic exosomes on miR-223 *Grin2b* and *Gria2* expression are also observed in mouse neuronal cultures, we added astrocytic exosomes in mouse cortical neuronal cultures. We found increased levels of mouse neuronal miR-223 and reduced mouse neuronal Grin2b mRNA and protein expression (via immunostaining), as well as reduced *Gria2* mRNA expression, suggesting a conserved effect between species (Fig. 5f–i). In order to determine if the increased expression of miR-223 in astrocytic exosomes is necessary to induce reductions in neuronal *Grin2b* and *Gria2* expression, we inhibited miR-223 in astrocytes via locked nucleic acid (LNA) miRNA inhibitors and extracted exosomes. We found that inhibition of miR-223 in astrocytes was sufficient to rescue the exosomal-mediated deficits in *Grin2b* but not *Gria2* mRNA expression (Fig. 5h, i). These data suggest that exosomal-mediated reductions in *Grin2b* expression are mediated by miR-223, whereas additional exosomal miRNAs or mRNAs are needed for miR-223 to regulate *Gria2* expression in neurons. We conclude that mouse astrocytic exosomal miR-223 is assimilated by cortical neurons and is sufficient to inhibit neuronal *Grin2b* expression.

## Discussion

Mounting evidence has linked glutamatergic synaptic transmission and GABAergic and inflammatory gene expression in SCZ and BD [2, 3, 10–23]. Here we show that miR-223, a known exosome-enriched miRNA, is significantly increased in the OFC of subjects with SCZ and BD with psychosis, and is negatively correlated to the mRNA levels of its targets *GRIN2B* and *GRIA2*, which are also significantly downregulated. Moreover, we find that changes in miR-223 in the OFC of BD and SCZ patients are positively and negatively associated with alteration in *SERPINA3* and *GAD1* mRNA expression, respectively. Furthermore, reductions in miRNA-processing inhibitors ADAR1/2 also display a negative correlation with miR-223, which is unaltered at the pri-miRNA level. In addition, we show that miR-223 is highly expressed in astrocytes, and is enriched in cortical glial and neuronal exosomes, and that antipsychotic treatment regulates cellular and exosomal miR-223 abundance in a cell-specific manner. Lastly, we demonstrate that addition of astrocytic exosomes containing miR-223 to cortical neuronal cultures results in increased neuronal miR-223 expression and reductions in neuronal *Grin2b*, which are corrected following inhibition of miR-223 in astrocytes. Taken together, our data suggest that a glia-enriched exosome-secreted miRNA regulated by antipsychotics is dysregulated in the frontal cortex of subjects with psychosis and associated with alterations in glutamate receptor gene expression.

Previous work has shown that miR-223 specifically controls Grin2b and Gria2 protein levels in mouse hippocampal neurons by directly repressing their expression through binding to highly conserved between mouse, rat, and human complementary mRNA sites in their 3′UTRs [50]. The same study also found that overexpression of miR-223 resulted in reduced NMDA-mediated calcium influx and miniature excitatory postsynaptic currents in hippocampal neurons, and that hippocampal miR-223 was an important modulator of contextual memory [50]. An additional study from the same group also found that inhibition of miR-223 in human stem cell-derived neuronal cultures increased NMDA-induced calcium influx, suggesting a conserved action of miR-223 on NMDA-mediated synaptic function [56]. It is tempting to hypothesize that the observed upregulation of miR-223 could contribute to chronic NMDA receptor hypofunction and reduced synaptic activity, a proposed  component of SCZ pathophysiology [3, 4]. Furthermore, previous studies have suggested that miR-223 is an inflammation-induced miRNA that acts to limit inflammation by targeting numerous immune-related molecular nodules [70–72]. Given the positive correlation between miR-223 and inflammation-associated astrocyte-enriched *SERPINA3*, which was also shown to be increased in both SCZ and BD patients with psychosis, it is possible that inflammation and glial activation could contribute to the expression of miR-223 in the OFC. Additional work is needed to determine the effects of neuroinflammation on miR-223 alterations in psychiatric disorders.

For miR-223 to be able to influence both glial and neuronal gene expression, it is imperative that it is either abundantly expressed in all brain cell types or that it can be transferred from one cell type to the other. Our data suggest that miR-223 is enriched in astrocytes, but displays moderate expression in neurons. In accordance with numerous papers showing miR-223 to participate in cell-to-cell communication through its secretion via exosomes [48, 49], our data suggest that secretion of miR-223 from astrocytes allows it to regulate NMDA receptor gene expression in recipient neurons. Of note, a recent study that screened for miRNAs that are enriched in exosomes suggested that miR-223 was among the most enriched and efficiently packaged in exosome miRNAs, with very low abundance outside of exosome fractions [48]. On the other hand, having miR-223 secreted from peripheral monocytes and macrophages, might allow some peripherally generated mature miR-223 molecules to cross the blood–brain barrier (BBB) and influence brain gene expression, especially in cases where the BBB permeability is increased. Future studies are needed to determine the exact balance of peripheral and central miR-223 production in response to inflammation.

Although our study focused on miR-223, additional upregulation in SCZ and BD OFC miRNAs observed in our study could be of relevance to psychiatric disorders. For example, miR-193a-3p, a miRNA of the same family to miR-193b-3p, which is upregulated in both BD and SCZ in our study, was found to be a reliable blood biomarker in a study with a large number of patients with SCZ [73]. On a similar note, miR-330-3p, which is increased in the OFC of BD patients in our study, has also been found to be increased in the blood of subjects with BD and monopolar depression [74]. Moreover, miR-28a-3p, also increased in our study, is in the same miRNA family as miR-708, a miRNA shown to be linked to BD in GWAS analyses [75].

One of the limitations of our study is that we did not measure miRNA expression in postmortem samples from other major brain regions significantly impacted in SCZ, such as the dorsolateral prefrontal cortex (DLPFC). However, miR-223 has been shown to be among the many upregulated in SCZ DLPFC miRNAs in a previous study [37], suggesting the possibility that is altered in numerous brain regions. Another limitation of our study is that the significant reported alterations in our NanoString miRNA profiling cannot survive correction for multiple comparisons and demographics. However, using mature miRNA-specific qRT-PCR, which is more accurate than any screening method, we have validated the changes shown in the screen following statistical correction for multiple postmortem demographics. Moreover, given the differences in structure, function, and expression of primary neuronal and glial cultures in comparison with neurons and astrocytes in the brain, the observed differential effects on the expression and exosomal secretion of miR-223 in mouse neuronal and astrocytic cultures following short-term treatment with antipsychotics cannot be extrapolated to occur in the brain. Additional in vivo studies are needed to further explore the influence of antipsychotics on miR-223 neuronal and glial cellular and exosomal expression. Furthermore, although miR-223 expression in human OFC was not significantly associated with overall duration of antipsychotic treatment in our cohort (shown as mg of fluphenazine equivalents), we cannot confidently assume that antipsychotic treatment has no effects on miR-223 expression. Indeed a previous study in mice has shown that although chronic treatment with haloperidol or clozapine does not affect the expression of miR-223 in the brain, olanzapine treatment can result in a significant reduction in brain miR-223 levels [59]. It is, therefore, possible that the observed increase in miR-223 expression in the OFC of subjects with SCZ or BD with the presence of psychosis at the time of death could be ameliorated in a subset of patients that were treated with olanzapine. Future work in additional cohorts that contain more detailed information on the type and duration of antipsychotic treatment is needed to fully assess their impact on miR-223 expression in the OFC.

## Funding and disclosure

This work was supported by a mentored PI grant as part of a P20 grant from the NIGMS (1P20GM121176-01—N.M. and S.K.A.). We would like to thank the SMRI brainbank for providing us with postmortem brain specimen, Colleen Ramsower and Crystal Richt at the University of Arizona Genetics Core for assistance in miRNA profiling, Mathew Campen and Tamara Young for help with NanoSight, Tamara Howard for EM assistance, Evelyn Lozano, and Eunice Amoah for technical assistance, and Nora Perrone-Bizzozero, Erin Milligan, Juan Bustillo, and Andrea Allan for useful advice and feedback. This work was supported in part by Dedicated Health Research Funds from the University of New Mexico School of Medicine (N.M). The authors declare no competing interests.

## Supplementary information

Supplementary figures and methods

APC Form
