# Supplementary material for: Exosomal secretion of a psychosis-altered miRNA that regulates glutamate receptor expression is affected by antipsychotics
Source: Neuropsychopharmacology. 2019 Nov 27;45(4):656–65. doi: 10.1038/s41386-019-0579-1 (PMC7021900; doi:10.1038/s41386-019-0579-1)
Supplement: Supplementary file 1 — Supplementary figures and methods [file 41386_2019_579_MOESM1_ESM.docx]

**SUPPLEMENTARY MATERIAL**

**Exosomal secretion of a psychosis-altered miRNA that regulates glutamate receptor expression is affected by antipsychotics**

Stephen K. Amoah^1,2^, Brian A. Rodriguez^1^, Constantine N. Logothetis^1^, Praveen Chander^1^, Carl M. Sellgren^3^, Steven D. Sheridan^4,5^, Jason P. Weick^1^, Lauren L. Jantzie^6^, Maree J. Webster^7^, **Nikolaos Mellios**^1,2#^

*^1^Department of Neurosciences, University of New Mexico School of Medicine, Albuquerque, NM, USA.* *^2^ Autophagy inflammation and metabolism (AIM) center, Albuquerque, NM, USA. ^3^Department of Physiology and Pharmacology, Karolinska Institutet, Stockholm, Sweden. ^4^Center for Genomic Medicine, Chemical Neurobiology Laboratory, Departments of Neurology and Psychiatry, Massachusetts General Hospital and Harvard Medical School, Boston, MA, USA. ^5^Harvard Medical School, Department of Psychiatry, Boston, MA, USA; Center for Experimental Drugs and Diagnostics, Center for Human Genetic Research, Massachusetts General Hospital, Boston, MA, USA. ^6^Department of Pediatrics, Johns Hopkins School of Medicine, Baltimore, MD, USA. ^7^Laboratory of Brain Research, Stanley Medical Research Institute, Chevy Chase, MD, USA.*

*^#^To whom correspondence should be addressed – Nikolaos Mellios, University of New Mexico School of Medicine, Department of Neurosciences, BMSB 145, 915 Camino de Salud NE, Albuquerque, 87131, NM. Email:* [*nmellios@salud.unm.edu*](mailto:nmellios@salud.unm.edu)*. Tel: 505-272-8451.*

**Table of Contents:**

Page 2-4: Supplementary methods and materials and related references.

Pages 5-10: Figures S1-5 and related figure legends.

**METHODS AND MATERIALS**

**Primary cultures.** Mouse cortical astrocyte and neuronal cultures were derived from the cortices of P0-P1 C57BL/6 male and female pups (The Jackson Laboratory, Bar Harbor, ME), respectively and were established according to previously published method [1-2]. Rat primary cortical neurons were derived from Sprague Dawley E18 male and female rats (Gibco/ThermoFisher Scientific, Waltham, MA). Both mouse and rat cortical neuronal cultures were cultured as described previously [2]. Brain dissection in P0-P1 C57BL/6 pups was performed in ice-cold Hanks’ balanced salt solution (HBSS) solution (Sigma Aldrich, St. Louis, MO) supplemented with the following: 20% FBS and NaHCO3 (4.2mM), HEPES (1 Mm; Sigma); pH 7.4. Dissected cortex, excluding hippocampus, cerebellum and brain stem was digested for 10 min with 0.25% trypsin (ThermoFisher Scientific). Tissue was washed and dissociated with flame-polished Pasteur pipettes of decreasing diameter in ice cold HBSS containing DNase (1500 U; Sigma). The cells were pelleted, resuspended in plating media and plated at a density of 4-5 x 10^4^ cells/12-mm coverslip coated with poly-Ornithine (0.1 mg/ml; Sigma, catalog #4638) and laminin (5ug/ml; ThermoFisher Scientific). Cells were allowed to adhere in 20 min before addition of 500µl plating media containing Neurobasal supplemented with 1x B27 plus, 2mM Glutamax 0.5 mg/ml Penicillin/Streptomycin/Neomycin (PSN) (all from ThermoFisher Scientific). In parallel, embryonic day 18 rat cortical neurons (ThermoFisher Scientific) of 2x10^4^ density were plated recapitulating the steps used for the primary mouse neurons. Serum was eliminated from the media after 24 h and again replaced after 48 h supplemented with 4 µM cytosine 1-β-D-arabinofuranoside (Ara-C; Sigma). Neurons were fed by replacing half the volume of spent media with fresh media without serum or Ara-C every 5 days. Primary mouse neurons and rat cortical neurons were grown for 18 and 12 days, respectively. For primary astrocytic cultures cells were pelleted, resuspended in DMEM supplemented with 10% FBS and 10% 1x PSN plated at a density of 1 x 10^6^ cells/Tissue Culture plate (ThermoFisher Scientific). The cells were plated in a Tissue culture plate and grown for 5-7 days until 90% confluent, then passaged using 0.25% trypsin (ThermoFisher Scientific). The passaged astrocytes were split into 4 tissue culture plates and grown for 10-14 days until 90% confluent exosomes were generated from the conditioned media. The astrocyte-conditioned media (ACM) was changed every 5 days and stored in -80^0^C and the astrocytes were split between 5-15 days following seeding when the cells achieved 90% confluence.

**Exosome generation and utilization.** Primary astrocytes were seeded into 24-well plates and after 1 day were subjected to the following treatments: olanzapine (100nM), haloperidol (100nM), (Sigma-Aldrich), or equivalent volume of vehicle (0.08 % ethanol) and were cultured for an additional 2 days. The same treatments and duration of treatment were performed in day 18 mouse primary cortical neuronal cultures. The stored astrocyte conditioned media (ACM) and neuron-conditioned media (NCM) were thawed and centrifuged at 2000g for 30 minutes to remove cells and debris, then Total Exosome Isolation buffer (ThermoFisher Scientific) corresponding to one half of the volume of the media were added i.e. 1.5mL ACM and NCM had 0.75mL, while 30mL ACM had 15mL of the buffer added. The media and buffer mixture was briefly vortexed and kept in 4^0^C overnight. The next day, the media and exosome isolation buffer was spun (without vortexing) at 10,000g at 4^0^C for 1 hr. Exosomes generated from 24mL of astrocytic media were measured with NanoSight NS300 (Malvern Panalytical, Malvern, UK) and assessment using zeta potential nanoparticle tracking analysis (NTA) software was performed to determine the quantity and sizes of the exosomes defined within a range of 30-150 nm. To treat rat neuronal cultures with mouse astrocytic exosomes, exosomes were derived from 0.8-6.4mL mouse ACM and diluted in 50 µl neuronal media were added to 2x10^4^ rat or mouse cortical neurons in each well of the 24 well plates. Exosomes were serially diluted in 50 µl neuronal media and added to neurons, and incubated for 1-2 days - in exosome-depleted FBS and 10% penicillin, streptomycin and neomycin (PSN) (ThermoFisher Scientific). Vehicle-treated neuronal cultures received 50ul neuronal media without any exosomes. Following either 1 or 2 days of incubation the media was removed and the neurons were carefully washed and neuronal pellets were harvested tor RNA extraction and analysis.

**Electron microscopy of exosomes.** Grids with carbon films were glow-discharged for 30 seconds. Grids were floated face-down for 35 minutes on 10 µl drops of sample 0.1x sample diluted in PBS, then washed in 3 drops of ultrapure water. Excess liquid was wicked off onto filter paper, then the grids were stained for 1.5 minutes on 10 µl droplets of 2% uranyl acetate (aq.). Stain was cleaned and the grids air-dried.

**NanoString miRNA profiling.** For mature miRNA profiling the NanoString nCounter system miRNA Expression Assay Kit (NanoString, Seattle, Washington, USA) was utilized at the University of Arizona Genetics Core per vendor’s instruction and as shown before [3]. Normalization (utilizing the geometric mean of all miRNAs) and data analysis were performed using nSolver software (NanoString).

**miRNA expression in human microglia and monocytes/macrophages.** We pooled NanoString nCounter miRNA data related to human miR-223-3p expression in purified human adult brain microglia, immortalized microglia, and human monocytes and macrophages from a previously published report [4].

**Locked nucleic acid (LNA)-mediated miRNA Inhibition.** To inhibit miR-223 in mouse cortical astrocytes we added miRCURY® power LNA® miRNA inhibitors for mouse miR-223 (Qiagen, Hilden, Germany) without transfection reagents and at a concentration of 25 pmol per the manufacturer’s instructions.

**Immunohistochemistry.** Immunocytochemistry was performed as previously described [5]. Primary antibodies consisted of polyclonal anti-Grin2b antibody (#ab65783, Abcam, Cambridge, UK, 1:200) and polyclonal anti-MAP2 (#822501, BioLegend, San Diego, CA, 1:2000). Secondary antibodies DyLight 488 (1:1000; Thermo Fisher Scientific) and 680 (1:1000; Li-Cor, Lincoln, NE), were used. Image acquisition was performed with a spectral Leica TCS SP* confocal microscope and ImageJ was used for image analysis and quantification of Grin2b staining intensity in MAP2+ neurons (3-4 replicates per coverslip were used for each biological replicate).

**Statistical Analysis.** For postmortem measurements, a Univariate General Linear Model which corrects for RIN, Brain pH, PMI, and Refrigeration Interval was chosen (IBM SPSS Statistics 24 – IBM, Armonk, New York), given the normal distribution of values as assayed by D'Agostino-Pearson (omnibus K2) test (GraphPad Software, La Jolla, CA). Normalized values were divided to the mean of each control group and the relative to control miRNA/mRNA ratios were plotted as means ± SE using GraphPad Prism after removing up to 2 outliers using iterative Grubbs test (GraphPad Software). In all other comparisons between two groups a two-tailed one sample *t*-test was used. For correlations Spearman’s correlation coefficients and two-tailed p-values were calculated. Correlations were considered strong when they resulted in statistical power of more than 0.80.

**SUPPLEMENTARY REFERENCES**

1. Schildge S, Bohrer C, Beck K, Schachtrup C (2013): Isolation and culture of mouse cortical astrocytes. J Vis Exp 71:e50079.

2. Weick JP, Groth RD, Isaksen AL, Mermelstein, PG (2003): Interactions with PDZ proteins are required for L-type calcium channels to activate cAMP response element-binding protein-dependent gene expression. J Neurosci 23:3446-3456.

4. Mellios N et al. MeCP2-regulated miRNAs control early human neurogenesis through differential effects on ERK and AKT signaling. *Mol. Psychiatry* 2017; e-pub ahead of print 25 April 2017; doi: 10.1038/mp.2017.86.

4. Sellgren CM, Sheridan SD, Gracias J, Xuan D, Fu T, Perlis RH, *et al.* (2017): Patient-specific models of microglia-mediated engulfment of synapses and neural progenitors. Mol Psychiatry 22: 170-177.

5. Weick JP et al. Deficits in human trisomy 21 iPSCs and neurons. *Proc. Natl. Acad. Sci. U. S. A.* 2013; **110**: 9962-9967.

**
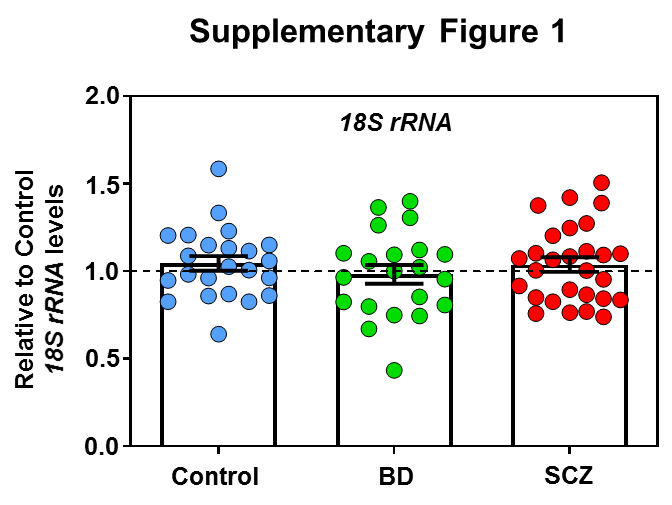
**

**Fig. S1. Expression of *18S rRNA* in BD and SCZ OFC.** Graph showing mean ± SEM relative to the mean of unaffected controls mRNA levels in SCZ, BD, and Control OFC for *18S rRNA* (Based on qRT-PCR in 20 fold diluted cDNA and without normalization). Data from each case are also depicted in the graph as blue circles (Control), green circles (BD), and red circles (SCZ).


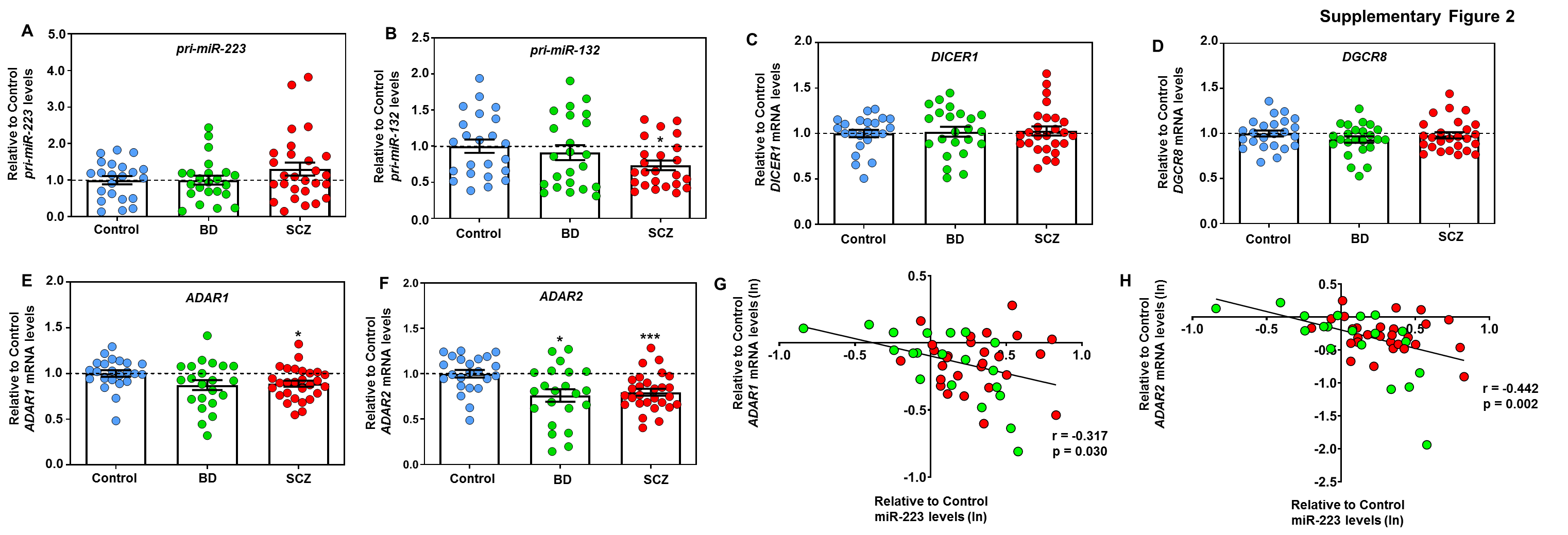


**Fig. S2. Expression miRNA biogenesis-associated genes and pri-miR-RNAs in the OFC of SCZ and BD patients.** **a-f** Graphs showing mean ± SEM relative to the mean of unaffected controls mRNA levels in SCZ, BD, and Control OFC for *pri-miR-223* (**a**) *pri-miR-132* **(b)**, *DICER1* **(c)**, *DGCR8* **(d)**, *ADAR1* **(e)**, and *ADAR2* **(f)** mRNA expression, based on qRT-PCR with the unaltered in SCZ and BD *18S rRNA* as a normalizer (see also methods and materials). *p < 0.05, ***p < 0.001, based on a univariate general linear model which corrects for RIN, Brain pH, PMI, and Refrigeration Interval (see also methods and materials). **g-h** Correlations between changes in miR-223 and *ADAR1* **(g)** and *ADAR2* **(h)** mRNA expression in the OFC of subjects with SCZ and BD. Spearman’s correlation coefficients and two-tailed p-values are shown in the graph. Relative to Control individual data are also shown in the graphs: SCZ = red circles, BD = green circles.

**
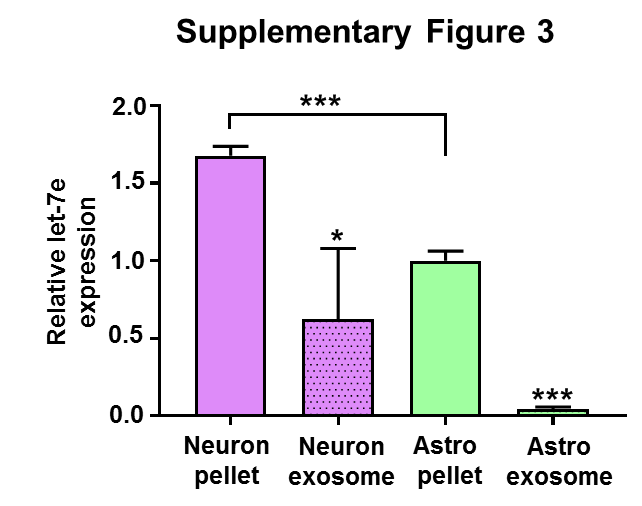
**

**Fig. S3. Cell specificity of mouse let-7e expression.**  Mean ± SEM let-7e relative expression in mouse cortical neuronal (2 weeks of differentiation) and astrocytic pellets and exosomes (based on mature miRNA qRT-PCR without normalization and shown as ratios relative to the highest expression in mouse astrocytic pellets). *p < 0.05, ***p < 0.001, based on two-tailed one sample *t-*test compared to pellet expression of the same cell culture (stars above bars) or compared to pellet expression between different cell types (stars with connecting line).

**
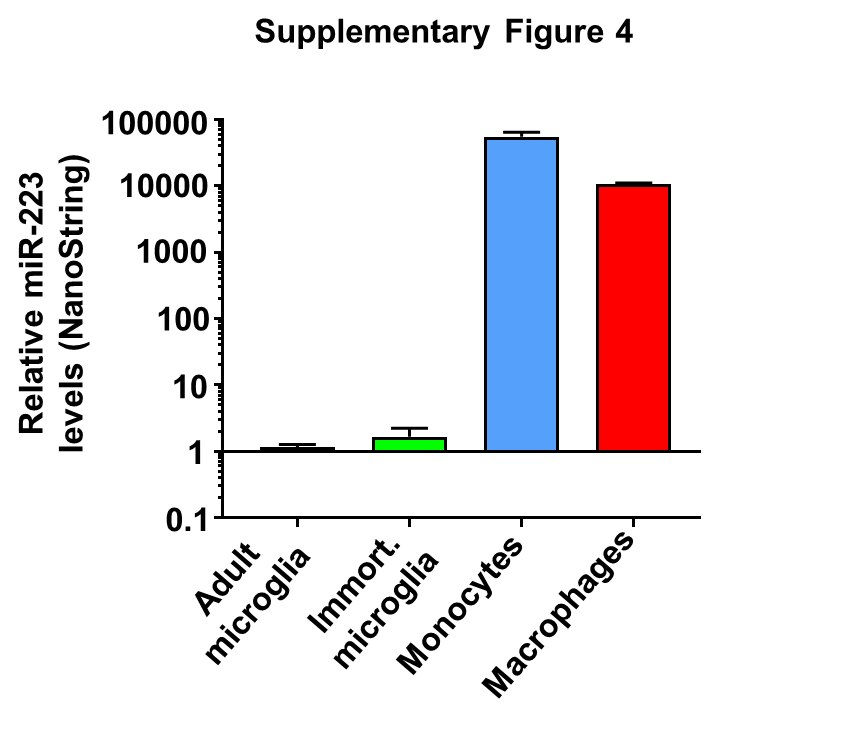
**

**Fig. S4. Expression of miR-223-3p in human microglia and peripheral monocytes and macrophages**. Mean ± SEM relative miR-223 expression in human adult (N=4) and immortalized (Immort., N=2) microglia, as well as peripheral monocytes (N=4), and macrophages (N=2), based on Nanostring miRNA profiling (see also methods and materials).

**
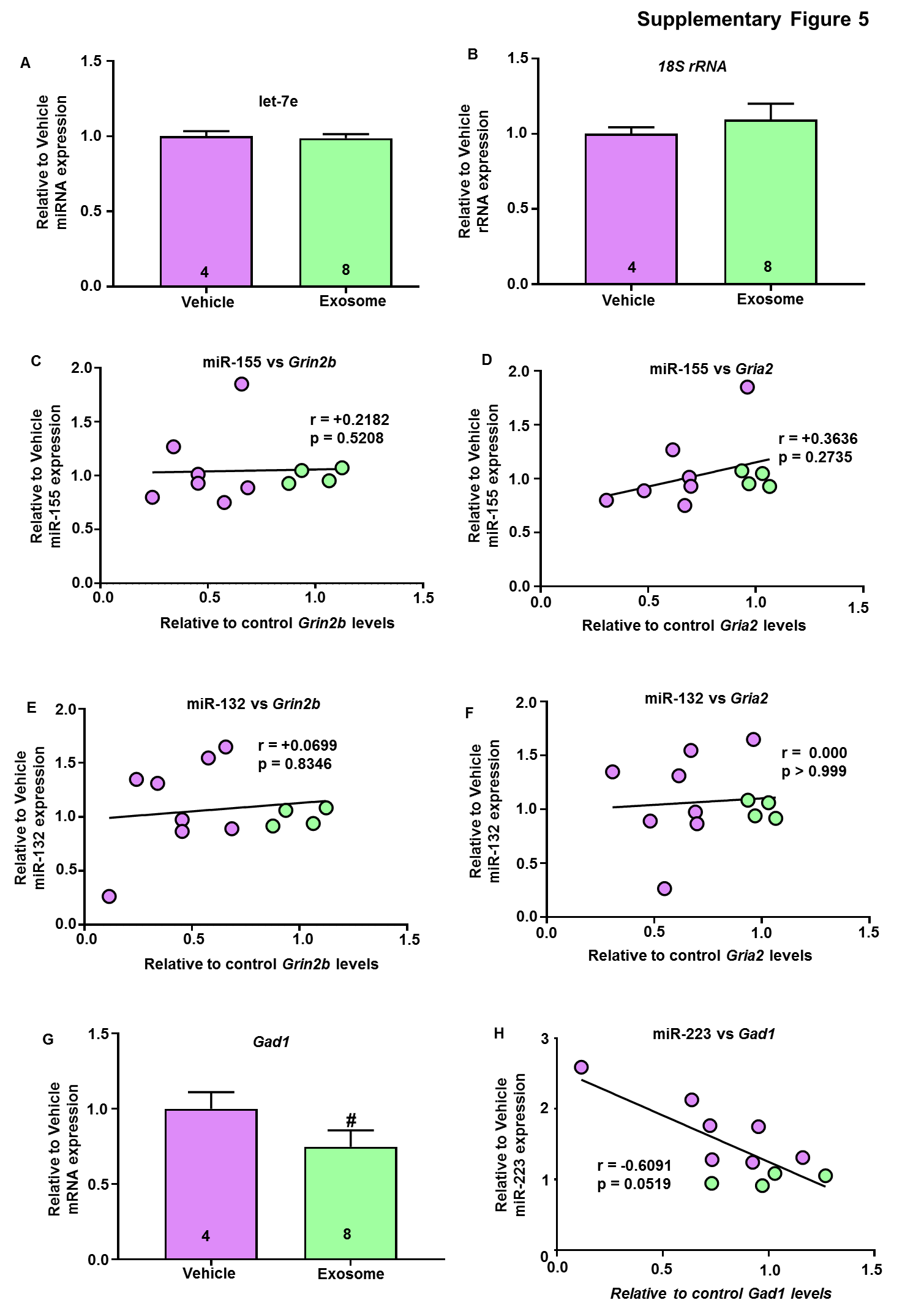
Fig. S5. Expression of let-7e, *18S rRNA*, and *Gad1* mRNA levels in exosome-treated rat cortical neuronal cultures and correlations between miR-132/miR-155 and *Grin2b/Gria2* mRNA expression. a-b** Graph showing mean ± SEM mature let-7e **(a)** and *18S rRNA* **(b)** expression (based on qRT-PCR without normalization) in rat neurons treated with mouse astrocytic exosomes relative to vehicle-treated rat neurons. **c-d** Correlations between relative to Vehicle miR-155 and rat *Grin2b* **(e)** and *Gria2* **(f)** mRNA expression **e-f** Correlations between relative to Vehicle miR-132 and rat *Grin2b* **(e)** and *Gria2* **(f)** mRNA expression. **g** Graph showing mean ± SEM rat neuronal gene levels following mouse exosomal treatment for *Gad1* mRNA (based on qRT-PCR and normalized to *18S rRNA*). Data are shown as ratios relative to the mean of Vehicle. **h** Correlation between relative to Vehicle miR-223 and rat *Gad1* mRNA expression. For **(c,d,e,f,h)** Spearman’s correlation coefficients and two-tailed p-values are shown in the graph. Vehicle = purple circles, Exosome treatment = green circles. For **(a,b,g)** ^#^0.05 < p < 0.10, based on two-tailed one sample *t*-test compared to Vehicle. The number of biological replicates is shown in each graph.
